# Gender differences in brain response to infant emotional faces

**DOI:** 10.1186/s12868-022-00761-5

**Published:** 2022-12-27

**Authors:** Kaihua Zhang, Xiaoyu Du, Xianling Liu, Wei Su, Zhenhua Sun, Mengxing Wang, Xiaoxia Du

**Affiliations:** 1 School of Psychology, Shandong Normal University, Jinan, 250358 Shandong China; 2 Faculty of Medicine, Dentistry and Health Sciences, The University of Melbourne, Victoria, 3010 Australia; 3 Department of Medicine Imaging, The People’s Hospital of Jinan Central District, Jinan, 250014 Shandong China; 4 School of Information Science and Engineering, Linyi University, Linyi, 276000 Shandong China; 5 College of Medical Imaging, Shanghai University of Medicine and Health Sciences, Shanghai, 201318 China; 6 Department of Psychology, Shanghai University of Sport, No.399 Shanghai Road, Yangpu District, Shanghai, 200438 China

**Keywords:** Gender differences, Infant emotional faces, Empathy, Functional connectivity, Functional magnetic resonance imaging

## Abstract

Infant emotional stimuli can preferentially engage adults’ attention and provide valuable information essential for successful interaction between adults and infants. Exploring the neural processes of recognizing infant stimuli promotes better understandings of the mother-infant attachment mechanisms. Here, combining task-functional magnetic resonance imaging (Task-fMRI) and resting-state fMRI (rs-fMRI), we investigated the effects of infants’ faces on the brain activity of adults. Two groups including 26 women and 25 men were recruited to participate in the current study. During the task-fMRI, subjects were exposed to images of infant emotional faces (including happy, neutral, and sad) randomly. We found that the brains of women and men reacted differently to infants’ faces, and these differential areas are in facial processing, attention, and empathetic networks. The rs-fMRI further showed that the connectivity of the default-mode network-related regions increased in women than in men. Additionally, brain activations in regions related to emotional networks were associated with the empathetic abilities of women. These differences in women might facilitate them to more effective and quick adjustments in behaviors and emotions during the nurturing infant period. The findings provide special implications and insights for understanding the neural processing of reacting to infant cues in adults.

**Supplementary Information:**

The online version contains supplementary material available at 10.1186/s12868-022-00761-5.

## Introduction

Adult and infant interaction relies heavily on the ability to receive and express nonverbal emotional signals through facial expressions. These nonverbal emotional signals, including infant emotional sounds or facial expressions, can attract the attention of adults and communicate their needs, thereby to obtain caregivers’ care and protection [1]. Human infants depend on sensitive and adaptive caregiving behaviors from adults for living [2]. Both women and men can provide caregiving behaviors for their infants. However, traditionally, women take responsibility for early childcare, whereas men have little direct investment in offspring [3]. Anthropological evidence has indicated that women are the primary caregivers of infants in the vast majority of cultures, whereas men are seen as more powerful and separated from the family [4]. Thus, biological or cultural factors (such as gender or social status) may produce the different parenting behavioral responsiveness to infants in women and men. Behavioral studies have indicated that women and men have different responses in the motivational processing of infants [5]. Women showed a greater preference for infants than men [6]. These parenting behavioral differences might be attributed to the different neural responsiveness between women and men. With regards to neural patterns in women and men response to infant cues, some studies have investigated brain processes in women and men in response to emotive sounds of infants. Independent of parental status (parent or non-parent), the brains of males and females reacted differently to infant cries, the results revealed that women interrupt mind-wandering when exposed to the sounds of infant hunger cries, whereas men carry on without interruption [7]. Seifritz et al. study showed that women but not men showed neural deactivation in the anterior cingulate cortex in response to both infant crying and laughing [8]. Messina et al. recorded motor evoked responses from arm muscles as produced by Transcranial Magnetic Stimulation in young adults [9] while listening to infant cries and found an excitatory modulation of MEPs at 100 ms from the onset of infant cry specific to females [9]. These investigations showed sex differences in brain responses to infant emotive sounds. Lorenz proposed the concept of Kindchenschema or ‘baby schema’ and suggested that the infant faces elicited a set of affective and behavioral responses that formed the foundation of caretaking behavior [10, 11]. The perceived sensitivity of caregivers in distinguishing between expression of emotional and neutral state signals in infants and responding appropriately and accurately may be considered an important prerequisite for establishing a ‘secure’ bonding [12]. Although the baby faces response is a fundamental social instinct that may be at the basis of human caregiving, its underlying neural mechanism is not well understood. we suspected that the specific brain circuits might also mediate women’s and men’s responsiveness to infant facial expressions, probably similar to results in women and men in response to infant sounds. Investigating individual differences in recognizing and responding to infant faces contribute to maternal sensitivity, which can profoundly influence later child development.

Infants usually communicate with adults by facial expression in the beginning of their lives. Thus, recognizing the emotion of the infant face can facilitate active human interactions. Studies indicated that the recognition of facial expression included several processes, such as processing the visual information and extract relevant features connected to the portrayed information, then infer the affective state based on these features [13]. Additionally, Kanske et al. reported that facial affect recognition combines the affection and cognition of social understanding, which enable such representations via creating vicarious affective states in the observer (empathy) [14]. Thus, we can utilize functional magnetic resonance imaging (fMRI) technology to explore the complex neural activation involved in generating and organizing responses to infant cues. For investigating the infant emotional faces evoked neural responses, several studies have begun to do so. Researchers have indicated that for adult women, in general and emotional infant faces, preferentially engage attention compared to adult faces. Therefore, infant faces may constitute a special class of social stimuli [15]. Recently, we also found that new mothers showed higher brain activation in regions involved in infant facial expression processing and empathic and mentalizing networks (e.g., inferior and middle frontal gyrus, middle temporal gyrus, lingual gyrus, fusiform gyrus, cuneus, parahippocampal gyrus, and middle and inferior occipital gyrus) than nulliparous women [16]. In these studies, they didn’t focus on gender differences in viewing infant emotional faces. It is widely assumed that women, and in particular mothers, show greater attunement to infants than do men [17, 18]. However, empirical evidence for gender effects, particularly in relation to perception of infant emotion using neuroimaging methods, has been lacking. An event-related brain potentials study was recorded in women and men when they observed infants’ faces, the results found gender differences including an asymmetric functioning of the visual cortex in men and a more bilateral functioning in women during decoding faces and expressions [19]. Existing evidence from this ERPs study might suggest that there are gender differences when it comes to processing social stimuli (faces and persons). Thus, understanding the different gender’s underlying neural mechanisms of processing infant emotional faces is contributing to understanding different gender’s established behavioral differences.

Prior neuroimaging studies mainly concentrated on exploring functional brain activity in response to infant stimuli using task-related fMRI. Task fMRI and resting-state fMRI (rs-fMRI) are twin techniques rooted on BOLD signal change and are effective predominantly because of their non-invasiveness. In comparison, the rs-fMRI method has gained advantages over task fMRI due to ease in signal acquisition, requisite of least effort from the subjects [20]. The spotlight of rs-fMRI is on the intrinsic activity within the brain in the absence of any cognitive stimulus. Greene et al. indicated that tasks have widespread effects on patterns of brain activity, of which focal task activations are only a small part, and that brain-phenotype relationships are best revealed by measures, such as functional connectivity (FC), that capture these distributed effects [21]. FC analyses, a family of methods investigating correlated activity between two or more brain regions, has exploded in popularity in recent years [22]. It offered sweeping insights into the macroscale neural circuits underlying complex cognitive processes, finding these circuits to be broadly distributed across the human brain [21]. FC could reveal “intrinsic connectivity networks” that recapitulate networks invoked during task execution [23]. Therefore, the analyses of FC in the state of rest could reveal different resting state networks, which depict specific functions and varied spatial topology. Here, the current study also acquires the rs-fMRI data; we defined the task-induced differential activation as regions of interest (ROIs) and explored the different patterns of resting-state FC between women and men using rs-fMRI data, that is, the current study also utilized the resting-state fMRI to focus on mapping functional communication channels between brain regions by measuring the level of correlated dynamics of fMRI time-series in adults.

Here, to identify the brain processing that underlies nulliparous women’s and men’s general propensity to respond to infant emotional faces, we recorded a task-fMRI from nulliparous women and men while they processed infant emotional faces. Further, differential brain regions in responses to infant emotional faces were selected as ROIs; then we used FC method to delineate their functional characterizations using the rs-fMRI data. Using a multimodal fMRI, we aimed at providing a comprehensive characterization of regions that showed differential brain activation responses to infant emotional faces by analyzing interactions of their functional connections. We specifically hypothesized that infant emotional expressions would activate adult brain regions critical for preparation for communicative, interactive and nurturing behaviors; these areas include the fusiform gyrus, cingulate cortex, parahippocampal gyrus, precuneus and inferior parietal lobe [24–26]. Furthermore, we predicted that the FCs in these regions in women were also different from men.

## Materials and methods

### Subjects

We recruited two groups of healthy volunteers by posting advertisements and social networks including 26 nulliparous women (between the age of 24 and 32 years old, M = 26.68, SD = 1.84) and 25 men (between the age of 24 and 32 years old; M = 26.54, SD = 1.92). All subjects without any neurological, medical, psychiatric condition and no history of severe head trauma were screened for scanning after giving written consent to participate. Strictly right-handed individuals were assessed by the Chinese Hand Preference Questionnaire that was written according to the Edinburgh Handedness Inventory [27].

All procedures were approved by the Ethical Committee of East China Normal University Committee on Human Research (No. HR201508001). Each volunteer signed an informed consent form that was approved by the committee. All methods in our study were carried out in accordance with the principles outlined in the Declaration of Helsinki, including any relevant details.

### Experimental materials and fMRI measurement

Before the experiment, we firstly selected a total of 120 color pictures of infant faces from the Chinese affective picture system [28]. These pictures included 40 happy infant faces, 40 neutral infant faces, and 40 sad infant faces. Every picture of size and background was unified to minimize any differences in stimuli’s physical characteristics. Then to exclude potential influence of other factors on brain activity, the pictures that were used in the scanner were selected within this database of 120 color pictures and rated by 29 adults (17 males, M = 25.10, SD = 1.89) on a 9-point Likert scale assessing arousal and valence. For arousal, the scale ranged from 1 (completely unaroused) to 9 (completely aroused), with a higher score meaning a higher arousal. For valence, the scale ranged from 1 (completely unhappy) to 5 (neutral) and to 9 (completely happy); The three groups of pictures differed significantly in each valence dimension [F = 91.13, p < 0.0001, Women, happy faces: M = 7.12, SD = 0.50; neutral faces: M = 5.14, SD = 0.35; sad faces: M = 2.63, SD = 0.60; Men, happy faces: M = 6.54, SD = 0.43; neutral faces: M = 5.12, SD = 0.29; sad faces: M = 2.84, SD = 0.45;]and arousal dimension [F = 16.80, p < 0.0001, Women, happy faces: M = 6.20, SD = 0.64; neutral faces: M = 3.50, SD = 0.89; sad faces: M = 6.60, SD = 0.80. Men, happy faces: M = 5.38, SD = 0.51; neutral faces: M = 2.87, SD = 0.68; sad faces: M = 6.09, SD = 0.60]. No significant differences were found between women and men in the arousal with FDR correction. They were recruited by public advertisement and participated in this behavioral experiment only. Each picture was presented for 3 s on a laptop randomly. Finally, we selected 60 stimuli including 20 happy, 20 neutral, and 20 sad faces from these 120 color pictures for the fMRI experiment. These pictures were balanced in arousal and valence. During the fMRI session, all subjects were presented with infant facial pictures in the scanner. Every picture was present with 2 s randomly, then followed by an average 4 s fixation cross (ranging from 2 s to 6 s). Every infant emotional face was presented twice, and the experimental paradigm was composed of 120 trials in total. This task has 363 volumes that last 12 min 6 s. All stimuli were presented using a SAMRTEC SA-9900 system (Shenzhen Sinorad Medical Electronics Inc., Shenzhen city, China). The SA-9900 system was used to provide synchronization between the stimuli presentation and the MRI scanner.

After the fMRI session, participants rated their arousal and valence while viewing a sample of 60 faces derived from the fMRI experiment stimuli. Each picture was projected on the screen for about 3 s in random order and participants subsequently rated its valence from “unhappy” to “happy” and its arousal from “calm” to “exciting,” using 9-point scales. The behavioral results were reported as follows: the valence and arousal of the internal consistency reliability coefficients examined in all infant emotional faces [valence: F (2, 59) = 125.53, p < 0.001, Cronbach’s Alpha = 0.89 and arousal: F (2, 59) = 58.55, p < 0.001, Cronbach's Alpha = 0.97]. Nulliparous women (happy: M = 7.59, SD = 0.86; neutral: M = 5.12, SD = 0.35; sad: M = 3.10, SD = 1.20) rated infant emotional faces more pleasant than men (happy: M = 7.26, SD = 0.75; neutral: M = 5.07, SD = 0.21; sad: M = 3.48, SD = 0.97), no significant differences were observed between women and men in feeling emotional infant faces with FDR correction. Nulliparous women (happy: M = 6.90, SD = 1.53; neutral: M = 3.11, SD = 2.01; sad: M = 6.68, SD = 1.50) also rated infant emotional faces more arousing than men (happy: M = 6.14, SD = 1.40; neutral: M = 2.93, SD = 1.65; sad: M = 6.36, SD = 1.22), no significant differences were found between women and men in the arousal with FDR correction.

All volunteers filled in the Interpersonal Reactivity Index (IRI) that used to assess participants’ empathic abilities [29, 30]. This IRI scale is a multi-dimensional assessment composed of 28 self-report items measuring four dimensions: The ‘empathic concern’ (EC) scale measures respondents’ prosocial feelings of warmth, compassion, and concern for others; The ‘personal distress’ (PD) scale measures self-oriented anxiety when observing others in distress; The ‘fantasy’ (FS) scale measures the tendency of the participant to identify with fictitious characters in books and movies; and the ‘perspective-taking’ (PT) scale assesses the tendency to take the psychological point of view of others. Studies reported that higher subscale scores are associated with higher empathic tendencies [30, 31]. The reliability of IRI analysis yielded high internal reliability coefficients with Cronbach’s Alpha = 0.86 in all subjects.

### MRI image acquisition

The MRI scanning was performed on a Siemens 3.0 T Trio Tim MR system at the Shanghai Key Laboratory of Magnetic Resonance (East China Normal University, Shanghai, China). We used a 12-channel head coil for the whole brain scanning. Anatomical images were collected using a high-resolution T1-weighted 3-dimensional magnetization-prepared rapid-acquisition gradient-echo pulse sequence with the following acquisition parameters: repetition time (TR) = 2530 ms, echo time (TE) = 2.34 ms, flip angle = 7°, inversion time = 1100 ms, acquisition matrix = 256 × 256 mm^2^, field of view (FOV) = 256 mm, 192 slices. A T2*-weighted gradient-echo echo-planar-imaging sequence, which is sensitive to blood oxygen level-dependent contrast, was used to collect functional images, with the following parameters: TR = 2000 ms, TE = 30 ms, FOV = 220 × 220 mm^2^, acquisition matrix = 64 × 64, 33 slices, slice thickness = 3.5 mm, 25% gap. During the resting-state fMRI scan, we acquired 210 whole-brain volumes with the following parameters: TR = 2000 ms, TE = 30 ms, flip angle = 90°, slices = 33, transverse orientation, FOV = 220 × 220 mm^2^, matrix size = 64 × 64, slice thickness = 3.5 mm, and 25% distance factor. All subjects were instructed to rest, relax, and not think of anything, with their eyes closed.

### MRI Data Analysis

#### Pre-processing of task-fMRI data

MRI data were analyzed with Statistical Parametric Mapping software (SPM12; http://www.fil.ion.ucl.ac.uk/spm/software/spm12) based on MATLAB 2015a. For data preprocessing: The preprocessing step contained slice timing correction, realignment, normalization to the Montreal Neurological Institute (MNI) space template, and spatial smooth was applied using a 4 mm Gaussian kernel. Slice timing correction was performed using the middle slice in time as reference. Spatial realignment was utilized to correct for head motion. The functional images were co-registered to the high-resolution T1-weighted images. The images were then spatially normalized to the MNI template (resolution of 3 mm × 3 mm × 3 mm) using the parameters obtained from segmentation. Finally, spatial smoothing with an 8 mm full-width half-maximum isotropic Gaussian kernel was performed on the functional images. Three subjects (one man and two women) were excluded because their translational head motion exceeded 2 mm or their rotational motion exceeded 2°. The remaining subjects entered the further statistical analysis.

#### Statistical analysis of task-fMRI data

After preprocessing, for each subject, images with an analytic design matrix were constructed, onsets and duration of each trial as epochs convolved with a hemodynamic response function used the general linear model in first-level statistical analysis. Six head movement parameters from realignment as regressions of no interest were also included. Three contrasts (happy, neutral, and sad) were designed. These images entered the second-level statistical analysis. To address our hypothesis of altered empathetic and reward processing in respond to infant emotional faces, we performed a 2 (group: nulliparous women vs. men) × 3 (condition: happy, neutral, and sad) ANOVA on whole-brain data using a flexible factorial model, with group as between-subject factor and emotional faces condition as within-subjects factor. Post hoc t-tests were conducted to further investigate potential interaction effects. The whole-brain significant results for functional imaging data were reported at the threshold of voxel level p < 0.001 and a cluster-size threshold of p < 0.05 corrected using family-wise-error (FWE).

#### Pre-processing of rs-fMRI data

The currents study also scanned rs-fMRI data. To examine whether differentially activated brain regions showing differences between nulliparous women and men in response to emotional infant faces acted in concert with other regions as a network, we performed FC analysis using rs-fMRI data. The resting-state data analysis was performed using DPABI [32]. The rs-fMRI data preprocessing applied in the research were as below: Original DICOM format was converted into NIFTI format; the first ten volumes of the functional images were discarded to allow for the magnetization to reach a steady state; the rest functional volumes were corrected with respect to slice timing and head motion correction by linear regression process, then images were realigned; structural images were co-registered to the average functional data, and normalized fMRI data were re-sliced with a resolution of 3 × 3 × 3 mm^3^; the processed data were smoothed with an 8-mm Gaussian kernel. The generated images were processed using linear trend removal and bandpass filter using 0.01 ~ 0.1 Hz. And several covariates of no interest were regressed from the data including head motion parameters, mean cerebrospinal fluid signal, mean white matter signal, and mean global signal. The residual time series of each subject was used to compute the resting-state FC.

#### FC analysis of rs-fMRI data

The preprocessed data was subjected to rs-FC analysis. Rs-FC maps for all subjects were obtained by calculating Pearson's correlation coefficient between ROIs and rest of the brain. First, the definition of ROIs: ROIs were defined from the significant differential brain regions in viewing infant emotional faces between women and men. The significant differential statistical parametric maps were from the above task-induced differential regions, these regions as seeds were explored to connect with the whole brain using FC method based on the rs-fMRI. The definition of ROIs used images calculator in SPM12. Then we computed the mean time series of the seeds and correlated these with the time series of other voxels in the whole brain to obtain FC maps. Finally, to improve the normality of the data distribution, FC maps were converted into z-score maps. Group effects were analyzed using the two-sample t-test in second-level statistical analysis. An initial threshold of p < 0.001 uncorrected was applied and results survived FWE correction at a cluster-level threshold of p < 0.05 were reported.

#### Assessment of IRI

All volunteers filled in the Interpersonal Reactivity Index (IRI) that used to assess a person’s empathy [33]. This IRI scale that is a persons’ multi-dimensional assessment of empathy consists of four sub-factors: cognitive empathy including Perspective Taking (PT) and Fantasy (FS); emotional empathy including Empathic Concern (EC) and Personal Distress (PD), respectively. Behavioral data of IRI are analyzed using SPSS 22 software with the independent sample t-test.

## Results

### Behavioural data of infant faces and IRI scores

Independent-sample t test was used to compare IRI scores between women and men. The results were shown in Table 1. Compared to men, nulliparous women showed significant differences in EC (p = 0.027). The EC subscale measures the respondents’ feelings of concern, warmth, and compassion for others. Hence, nulliparous women might have a stronger feeling of concern, warmth, and compassion for infants than men.

**Table 1:** Comparisons of the changes in IRI scores between nulliparous women and men

Dimensions	Nulliparous women	Men	T value	*p* value (two-tailed)
				Women *vs.* men
Number	26	25	–	–
Age	26.50 ± 1.88	26.72 ± 1.86	− 0.42	0.27
Education	15.81 ± 0.63	16.04 ± 0.84	− 1.12	0.68
EC	20.04 ± 3.74	17.44 ± 5.50	2.00	**0.03**
PD	13.60 ± 3.27	12.16 ± 4.11	1.40	0.09
PT	16.12 ± 3.94	16.24 ± 3.22	− 0.12	0.45
FS	15.70 ± 4.43	15.36 ± 4.47	0.24	0.41

This table showed differences in the age, education, and IRI scores between nulliparous women and men. Data are presented as the mean ± standard deviation (SD). Comparisons were calculated using independence sample *t-*tests, and results are reported at a significance level of *p* < 0.05

*PT* perspective-taking scale, *FS* fantasy scale, *EC* empathic concern scale, *PD*, personal distress scale

In bold: Significant difference as compared to men with p < 0.05

### Differential neural response to infant facial expressions compared nulliparous women to men

**Fig. 1:**
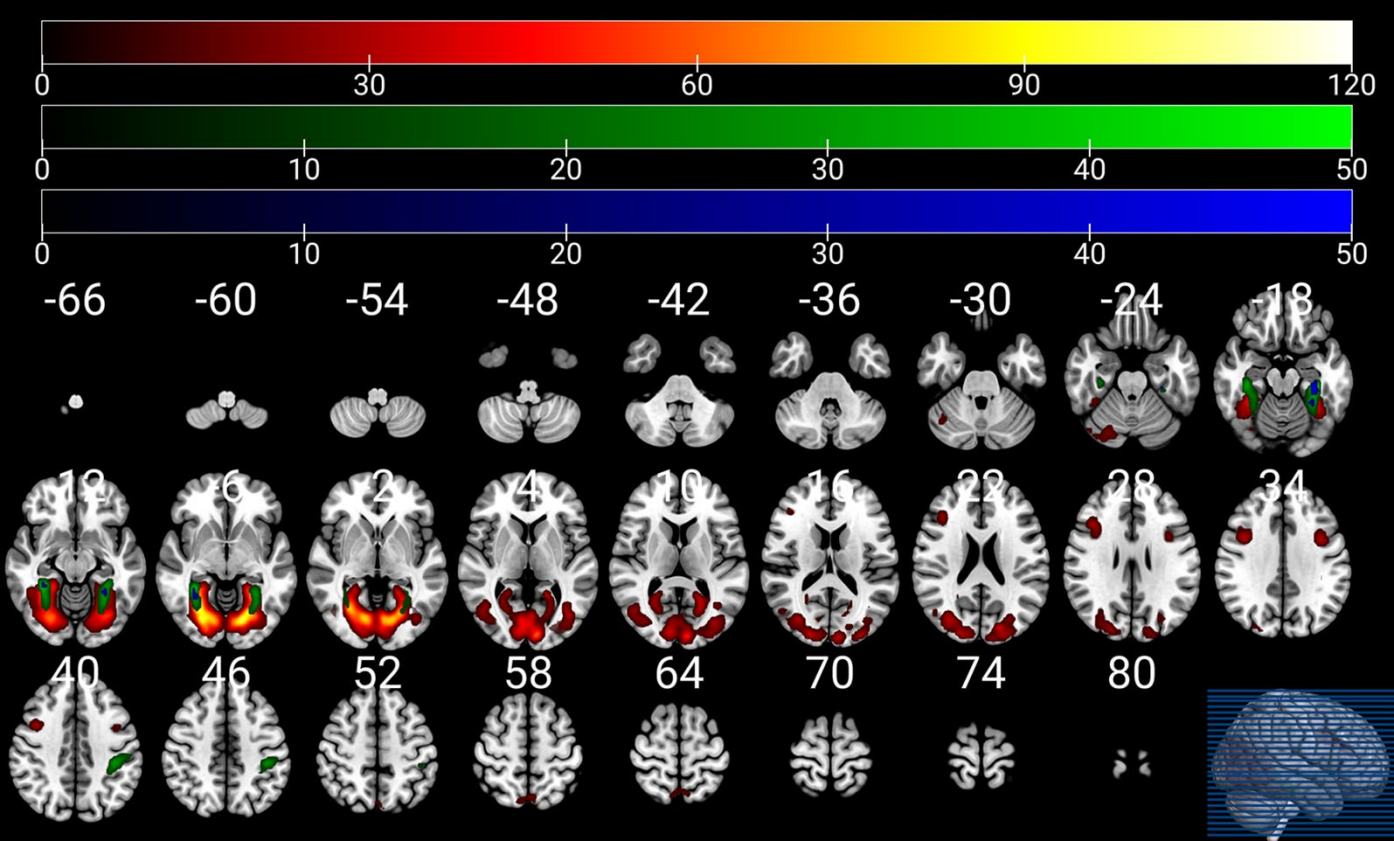
Brain regions with significant differential neural activations in response to infant emotional faces between nulliparous women and men. Red color: clusters showing a main effect of groups; Green color: clusters showing the interaction between group and condition for the happy vs. neutral contrast; Blue color: clusters showing the interaction between group and condition for the sad vs. neutral contrast

A main effect of groups was found in some clusters. Table 2 lists the location and composition of each cluster. The regions included the bilateral fusiform gyri, bilateral parahippocampal gyri, bilateral cuneus, bilateral superior and middle and inferior occipital gyri, bilateral posterior cingulate gyri, bilateral middle and inferior frontal gyri, bilateral precuneus, bilateral superior parietal lobule, bilateral cerebellum posterior lobe (Fig. 1 and Table 2). Follow-up t-tests revealed that the nulliparous women group had increased BOLD amplitude in these regions except the bilateral precuneus, bilateral superior parietal lobule, and bilateral cerebellum posterior lobe (see Additional file 1: Table S1).

**Table 2:** Functional brain imaging results for the main effects of groups and the group by infant emotional faces condition interaction with the post-hoc tests

Clusters	brain regions	BA	voxels	X	Y	Z	T	*P*^FWE*−corr*^
Main effect nulliparous women > men								
1	Bilateral lingual gyri	18/19/17/37/30/36	3497	30	− 63	− 6	11.02	0.001
	Bilateral cuneus							
	Bilateral fusiform gyri							
	Bilateral parahippocampal gyri							
	Bilateral middle occipital gyri							
	Bilateral inferior occipital gyri							
	Bilateral posterior cingulate gyri							
	Bilateral middle temporal gyri							
	Bilateral superior occipital gyri							
	Bilateral cerebellum posterior lobe							
	Bilateral precuneus							
	Bilateral superior temporal gyri							
2	Right middle frontal gyrus	9	199	39	21	27	6.08	0.001
	Right inferior frontal gyrus							
3	Left inferior frontal gyrus	9	89	− 39	6	33	5.64	0.025
	Left middle frontal gyrus							
4	Right thalamus	–	81	3	− 15	− 3	4.67	0.035
	Right brainstem							
5	Bilateral precuneus	7	79	3	− 72	51	− 3.16	0.038
	Bilateral superior parietal lobule							
6	Right cerebellum anterior lobe	–	97	24	− 75	− 24	− 3.16	0.018
Interaction group × condition (Happy vs*.* neutral faces)								
1	Left fusiform gyrus	37/36/19	275	− 33	− 30	− 18	6.94	0.001
	Left parahippocampal gyrus							
	Left cerebellum posterior lobe							
2	Right parahippocampal gyrus	37/19/36	244	33	− 45	− 9	6.34	0.001
	Right fusiform gyrus							
	Right cerebellum posterior lobe							
3	Left inferior parietal lobule	40/2	132	− 39	− 33	42	5.39	0.001
	Left postcentral gyrus							
Interaction group × condition (Sad vs*.* neutral faces)								
1	Left fusiform gyrus	37/36	224	− 30	− 0	− 21	6.63	0.001
	Left parahippocampal gyrus							
	Left cerebellum anterior lobe							
2	Right parahippocampal gyrus	19/37	207	33	− 45	− 9	5.90	0.001
	Right fusiform gyrus							
3	Left postcentral gyrus	40/2	99	− 39	− 30	42	5.01	0.017
	Left inferior parietal lobule							
4	Left superior parietal lobule	7	80	− 18	− 72	60	4.56	0.037
	Left inferior parietal lobule							
Interaction group × condition (Happy vs*.* sad faces)								
	None							

X, Y, Z = MNI coordinates. BA: Brodmann area. The results were reported at cluster level with *p* < 0.05 FWE correction at an initial uncorrected threshold of *p* < 0.001

Group-level statistical analysis identified clusters whose activity showed an interaction effect between condition and group. The group and condition revealed significant interaction effects in the bilateral fusiform gyri, bilateral parahippocampal gyri, bilateral cerebellum posterior lobe, left inferior parietal lobule, and left postcentral gyrus (Fig. 1 and Table 2). Follow-up t-tests analysis yielded a significant increase in BOLD contrast in nulliparous women compared to men for the happy versus neutral faces and sad versus neutral faces conditions in clusters again including the bilateral fusiform gyri, bilateral parahippocampal gyri, bilateral cerebellum posterior lobe, left superior and inferior parietal lobule, and left postcentral gyrus (Additional file 1: Table S1).

In addition, to rule out a potential contribution of structural differences between nulliparous women and men, mean gray matter values of each subject were added to the model as a nuisance regressor. The inclusion of structural data as a covariate didn’t change the pattern of our results (see Additional file 1: Table S2).

### Task-Induced functional connectivity analysis

**Fig. 2:**
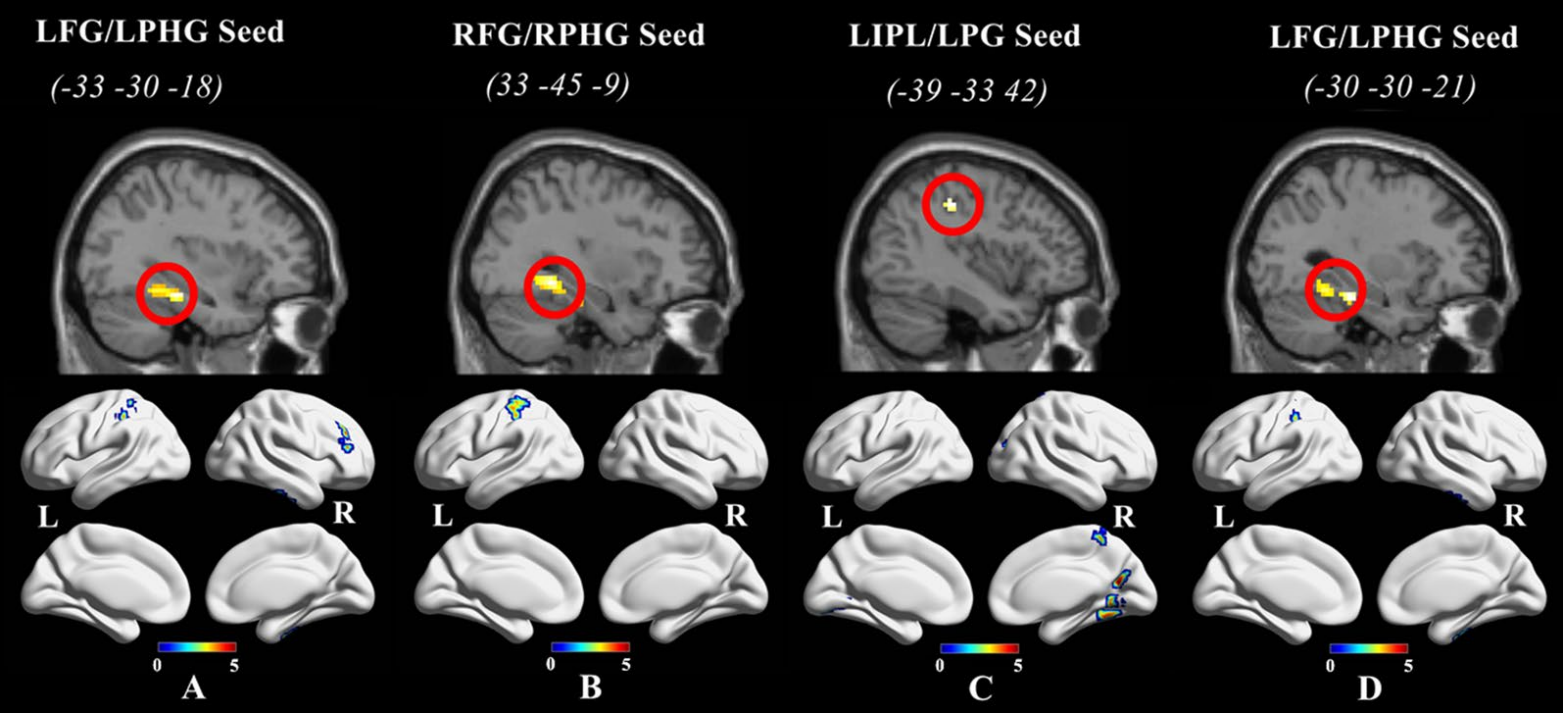
Significant increases in seed-based functional connectivity in nulliparous women compared to men. **A** seed-based resting-state FC for left fusiform gyrus (LFG) and left parahippocampal gyrus (LPHG); **B** right fusiform gyrus (RFG) and right parahippocampal gyrus (RPHG) for the seed-based analysis; **C** left inferior parietal lobule (LIPL) and left postcentral gyrus (LPG) for the seed-based analysis; **D** left fusiform gyrus (LFG) and left parahippocampal gyrus (LPHG) for the seed-based analysis; L: left, R: right

Results of the FC analysis showed significant alterations in nulliparous women and men between numerous pairs of regions, as shown in Table 3 and Fig. 2. When the significantly altered regions (e.g. the left fusiform gyrus and parahippocampal gyrus; the right fusiform gyrus and parahippocampal gyrus; the left inferior parietal lobule and postcentral gyrus;) determined in response to infant emotional faces were used as seeds, the between group comparison revealed significantly greater connectivity in nulliparous women compared to men in areas involving the right inferior temporal gyrus, right middle frontal gyrus, left cerebellum posterior lobe, left inferior parietal lobule, left postcentral gyrus, right precuneus, right middle occipital gyrus, right fusiform gyrus, right cerebellum anterior lobe (Table 3 and Fig. 2).

**Table 3:** Resting-state functional connectivity differences of nulliparous women compared to men

ROIs	Regions	BA	size	X	Y	Z	T value	*P*^FWE−corr^
Women > men								
Left fusiform gyrus	Right inferior temporal gyrus	20/	53	45	− 24	− 33	4.97	0.007
Left parahippocampal gyrus	Right middle frontal gyrus	9	46	33	36	33	4.50	0.015
	Left cerebellum posterior lobe	–	48	− 27	− 48	− 51	4.47	0.012
	Left inferior parietal lobule	40	65	− 36	− 39	42	4.36	0.002
	Left postcentral gyrus							
Right parahippocampal gyrus	Left postcentral gyrus	3/40	97	− 21	− 36	54	4.49	0.001
Right fusiform gyrus	Left inferior parietal lobule							
Left inferior parietal lobule	Right precuneus							
Left postcentral gyrus	Right cuneus							
	Right middle occipital gyrus	18	97	18	− 63	18	5.52	0.001
	Right paracentral lobule	7	36	15	− 48	60	5.07	0.043
	Right precuneus							
	Right fusiform gyrus		41	30	− 51	− 15	4.40	0.024
	Right cerebellum anterior lobe							
	Right lingual gyrus	19/18	54	15	− 57	− 3	4.35	0.006
	Right cerebellum anterior lobe							
	Left lingual gyrus	18	44	− 25	− 72	− 6	4.20	0.017
Left fusiform gyrus	Right inferior temporal gyrus	20	46	45	− 24	− 33	4.92	0.014
Left parahippocampal gyrus	Left cerebellum posterior lobe	–	82	− 27	− 48	− 51	4.85	0.001
	Left postcentral gyrus	40	61	− 24	− 33	60	4.55	0.003
	Left inferior parietal lobule							

This table indicated differences in functional connectivity in resting state in nulliparous women and men. X, Y, Z = MNI coordinates. BA: Brodmann area. The threshold was set at *p* < 0.001 uncorrected at the voxel wise level and *p* < 0.05 with FWE correction at the cluster level

## Discussion

In the current study, we combined a task-fMRI with an rs-fMRI to better understand how brain processing in response to infant emotional expressions differs between nulliparous women and men. As suspected, we found that compared with men, the neural activation in nulliparous women increased in several regions during the processing of infant emotional faces, such as visual areas (fusiform gyrus, inferior and middle occipital gyri, and lingual gyrus), limbic areas (parahippocampal gyrus, and posterior cingulate cortex), temporoparietal areas (parietal lobule, and middle temporal gyrus), temporal areas (middle temporal gyrus), and the cerebellum. This neural network is the most likely to be activated while processing the infant emotional faces. our findings indicated that processing emotion from infant facial expressions draws on diverse psychological processes, which is implemented in a large array of neural structures. We further investigated FC in resting-state networks that showed significant differential neural activation in response to emotional infant stimuli. Relative to men, some brain regions of the default model network (DMN) in nulliparous women showed increased connectivity (e.g., inferior parietal lobule, postcentral gyrus, precuneus, and middle temporal gyrus). Besides, the differential neural activations were associated with EC scores of nulliparous women. Therefore, these findings may provide new insights into the interface for the maternal brain responses to infant emotional expressions before nulliparous women becoming mothers.

In our task-fMRI study, women showed increased activation while viewing the infant emotional faces when compared to men, including the fusiform gyrus, parahippocampal gyrus, posterior cingulate gyrus, cuneus, middle temporal gyrus, middle and inferior frontal gyrus, inferior parietal lobule, middle occipital gyrus. We found higher activated regions in women than men response to infant emotional faces, consistent with the increased neural activation when mothers respond to infant-related stimuli [16]. These regions are related to the visual, emotional and rewarding processing. Specifically, greater activity in the fusiform gyrus was observed when test women viewed infant faces compared to men. Our results are consistent with previous studies, which demonstrated that the fusiform gyrus plays an important role in encoding the specificity of infant emotional faces [33–35]. The activity of the fusiform gyrus was modulated by individual differences in interest-in-infants [36], and the fusiform gyrus plays an important role in learning, monitoring, and memory of salient reward-related stimuli in the environment [37]. As the complement of the fusiform face area, the parahippocampal gyrus is related to the encoding emotional memory and retrieval [38]. Previous research has also shown the detection of biological significance is linked to emotional memory networks, which include the orbitofrontal cortex, parahippocampus, and hippocampus [39, 40]. There is some evidence to suggest that emotional cues are more easily memorized and recalled [41]. So, the emotional infant face might strengthen the memory and aid recall [42]. Another study also found that preterm infants could elicit more activity in the caudate, (para)hippocampi, and dorsomedial prefrontal cortex in mothers [43]. The posterior cingulate gyrus is a core region for the theory of mind or cognitive empathy (also called mentalizing) which requires cognitive reasoning to understand others, when compared to controls, postpartum women showed consistently more activated in this area when they saw babies, the findings suggest that posterior cingulate cortex might be a pivotal neural locus facilitating cognitive efforts to empathize with babies during the postpartum period [44]. As a key attentional control region involved in emotion regulation [45], the inferior parietal lobule is activated in the perception of emotions in facial stimuli [46], and is also a central node in the neural pathway of parental face perception [47]. Precuneus is involved in visuospatial processing, self-consciousness [48, 49] and activated when an individual makes a judgment that needs understanding whether to act out of empathy and forgiveness [50]. The regions of the lingual gyrus and cuneus, as parts of the medial occipital lobe, are necessary for both basic and higher-level visual processing [51]. A review study identified that emotional face-specific clusters were involved in face processing, including the fusiform gyrus, middle temporal gyrus, inferior frontal cortex [52]. Taken together, our results reflected that several brain regions related to functional neural networks in nulliparous women were activated when they responded to infant emotional faces. Moreover, these regions overlapped the neural basis of maternal behaviors, including maternal brain circuit, attention, facial visual cortex, and emotional modulation areas. The findings might suggest that complex infant cues require the allocation of attentional, empathetic, motivational resources in the brains of women to respond appropriately to them.

In our rs-fMRI study, to further confirm the gender differences between nulliparous women and men, we analyzed the resting-state networks that showed differences activation between women and men in response to emotional infant faces. Our FC resting-state results also demonstrated the functional connectivity of these areas (e.g., inferior parietal lobule, precuneus, postcentral gyrus, middle occipital gyrus, fusiform gyrus, and inferior temporal gyrus) increased in nulliparous women. Resting-state FC shown by a pattern of regions would result from habitual co-activation during goal-directed brain function [53]. Based on this, increased connectivity in these regions might be translated from inducing co-activation by the task of viewing infant's faces. We noticed that these regions are involved in hubs and subsections of a network known as the DMN [54–57]. For example, the precuneus was suggested as the ‘hub’ of the DMN that is activated when people do not engage intentionally in sensory or motor activity [56]. The medial temporal subsystem including parahippocampal gyrus and inferior parietal lobe are portions of DMN [55]. Normally, the DMN is involved in many functions, in addition to broadly monitoring the external environment, generate and manipulate mental images, remind of past experiences based on episodic memory, and make plans [58]. The cognitive performance was positively related to DMN functional connectivity [59, 60]. Additionally, Paola Rigo et al. have reported the similar results using the infant sound as stimuli, which infant sounds affect women and men differently [7, 61]; moreover, reduced rs-FC in DMN regions involved in social cognition was found in postpartum depression [62]. Thus, greater DMN functional connectivity in the current study might provide new insight for understanding the neural mechanism of parental rearing behaviors. Increased functional connectivity may help women respond to the infant cues necessary to facilitate proper caregiving behaviors. For example, when a woman becomes a mother, she can take appropriate maternal behaviors for caregiving by the reminiscence of past experiences based on episodic memory.

To examine the empathic capacities, we calculated the cognitive scales (including PT, FS) and the effective scales (including EC, PD) in all volunteers. We found a sex difference in the affective dimension of empathy (EC component), women reported higher EC scores than men in the current study, while there were no differences in cognitive empathy [63]. EC subscale measures women’s tendency to experience feelings of warmth, compassion, and concern for others. It is considered as affective empathy (feeling for) and is other-orientated and usually results in sustained functioning and helping behaviors [64]. Our results are consistent with previous research that there are differences in the capacity for empathy between males and females [63, 65]. Our results again proved that females have higher empathy levels than males [66, 67]. Women are more emotionally responsive and more likely to care for others than males and show more empathic responses neurologically when seeing others suffer [68]. As the primary caretakers of young infants, the stronger empathy abilities in women may facilitate sensitivity to infants’ internal states and resultant nurturing behavior [69]. These gender differences in foundational aspects of empathic behavior may derive from maternal care from an evolutionary perspective [69, 70]. Additionally, we made an exploratory analysis of the relationship between empathetic capacities and neural activation in nulliparous women and men (see Additional file). Empathy networks in the parental brain are involved in sensitive parenting behaviors [71], and the sensitivity of maternal behaviors contributes secure parent-infant attachment and the development of infant’s social cognitive functions [72]. Therefore, in the daily social environment, empathy abilities for caregivers provide adequate care for their children and successfully rearing offspring, specifically, empathy abilities can promote caregivers to understand their infants’ needs, decode social stimuli among others. In our results, increased empathic concern (EC score) and weaker activation and deactivation in nulliparous women respond to infants’ stimuli might suggest that women are more sensitive to infants’ expressions or sounds, and infants’ needs, thereby they can response to and deal with infants’ emergency events quickly and accurately. Thus, the enhanced ability to encode emotional faces in women may be an evolutionary adaptation, it can promote women to make preparations for the protective and nurturing demands of motherhood [73]. The capacity of caregivers for empathy is linked to the well-being and development of infants [4]. Thus, the findings suggested that nulliparous women with higher empathetic abilities may be more sensitive and easier understand the emotional infant cues.

Although our research revealed that women and men exhibited different brain activations in response to infant emotional faces, the current study had several limitations. First, the sample size was not sufficiently large, and more subjects and more observations are needed to verify the current results. Second, the gender of the infant facial stimuli was not considered. This factor may induce different feelings and affections in women and men. Third, the task should control for the confounding factors to confirm differences in empathy between men and women in perceiving social-emotional stimuli, rather than being specific to the emotional faces of infants, such as in the task, added adult faces might be better. Finally, the task design of 20 event-related trials per condition was weakly-powered within-subject.

## Conclusions

The present study intended to investigate the neural brain activations in nulliparous women and men in response to infant emotional expressions. Our results indicated that nulliparous women and men have a different activation pattern in empathetic and facial visual cortex related regions during viewing infant emotional faces. Furthermore, rs-fMRI indicated there are significant increases in FC of DMN in nulliparous women. Correlation analysis also showed that the differential empathetic abilities are related to neural activations between nulliparous women and men.

Our data suggest a gender difference in brain responses to infant emotional faces, especially regarding the activation of the facial processing, attention, and empathetic related system. Researchers have reported that sex differences in the frequency of nurturing behaviors [69, 74], in other words, males interacted less with the infant than did females. In childless adults, women showed relatively greater preferences for infants than men [75] which this kind of sex difference may represent a biological adaptation for parenting [6]. Hence, the differential brain function between nulliparous women and men may be explained to some extent on the cause of the behavior differences. These functional and behavioral differences in nulliparous women and men may promote females to adapt and adjust behaviors and emotions in favor of nurturing infants quickly when they experience pregnancy. These findings have implications for understanding the neural processing mechanism of nulliparous women and men respond to infant emotional faces.

## Supplementary Information

**Additional file 1**: **Figure S1. **Regions with correlations between brain differential activations (extracted beta values) and empathetic ability (EC scores) in nulliparous women and men. (A) associations between the clusters and EC scores in nulliparous women; (B) associations between the clusters and EC scores in men. **Table S1. **Functional brain imaging results for the main effects of group and the group by infant emotional faces condition interaction with the post-hoc tests. **Table S2. **Functional brain imaging results for the main effects of group and the group by emotional infant faces condition interaction (without grey matter volume as regression).

## Data Availability

The datasets generated during and/or analyzed during the current study are available from the corresponding author on reasonable request.
