# Supplementary material for: Gender differences in brain response to infant emotional faces
Source: BMC Neurosci. 2022 Dec 27;23:79. doi: 10.1186/s12868-022-00761-5 (PMC9793562; doi:10.1186/s12868-022-00761-5)
Supplement: Supplementary file 1 — Additional file 1: Figure S1. Regions with correlations between brain differential activations (extracted beta values) and empathetic ability (EC scores) in nulliparous women and men. (A) associations between the clusters and EC scores in nulliparous women; (B) associations between the clusters and EC scores in men. Table S1. Functional brain imaging results for the main effects of group and the group by infant emotional faces condition interaction with the post-hoc tests. Table S2. Functional brain imaging results for the main effects of group and the group by emotional infant faces condition interaction (without grey matter volume as regression). [file 12868_2022_761_MOESM1_ESM.doc]

**Additional file**

**Figure S1.**


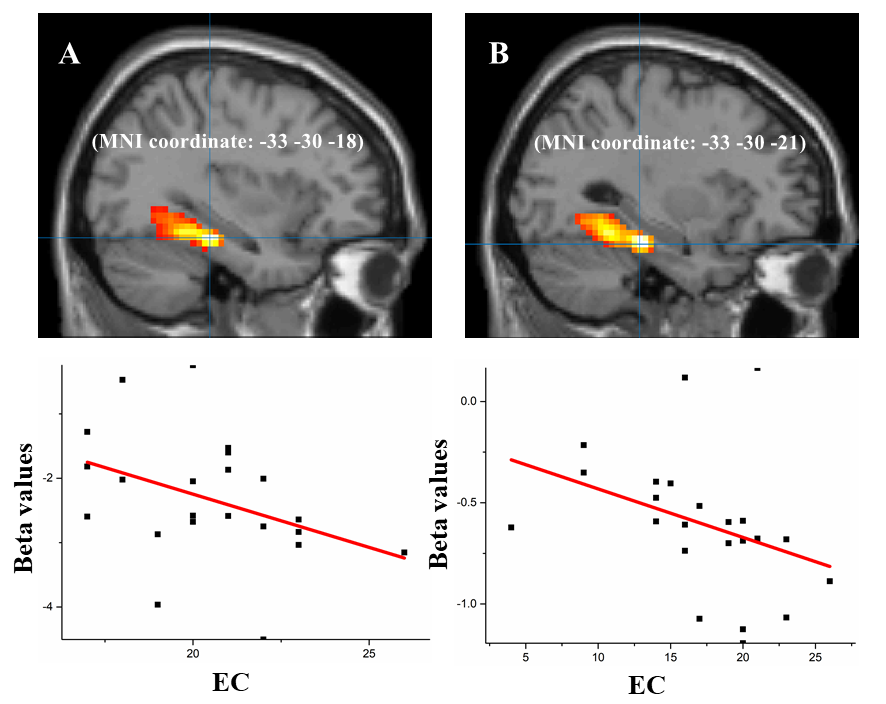


**Figure S1.** Regions with correlations between brain differential activations (extracted beta values) and empathetic ability (EC scores) in nulliparous women and men. (A) associations between the clusters and EC scores in nulliparous women; (B) associations between the clusters and EC scores in men.

**Exploratory correlation analyze:** To explore possible relationships between significant clusters of activation and IRI scores, an exploratory correlation analyze was performed in SPSS. Specific activations identified in the interaction between groups and conditions were used to define regions of interest (ROI). We extracted these individual mean beta values for Pearson correlation with IRI scores. SPM was used to define these ROI regions as masks, then the ROI signal extractor in DPABI was used for each ROI of every subject to extract mean parameter estimates for further correlation analysis in SPSS 22. The significant results were reported at p < 0.05 (two-tailed) (see Supporting Information **Figure S1**).

**IRI data associations with neural activation:** Correlation analysis between IRI scores and the brain activations extracted the result of between-group analysis (happy versus neutral) showed the activated cluster (peak MNI coordinate: -33 -30 -18) of nulliparous women included in the left fusiform gyrus, left parahippocampal gyrus and left cerebellum posterior lobe are negatively related to EC (r = -0.33, p = 0.04); However, when the between-group activations were extracted during viewing sad versus neutral faces as masks to be related to IRI, we found that the activated cluster (peak MNI coordinate: -30 -30 -21) including the left fusiform gyrus, left parahippocampal gyrus and left cerebellum anterior lobe have negatively associated with EC (r = -0.35, p = 0.02) in men.

**Table S1. Functional brain imaging results for the main effects of group and the group by infant emotional faces condition interaction with the post-hoc tests.**

| **Clusters** | **Brain regions** | **BA** | **voxels** | **X** | **Y** | **Z** | **F** | ***P*^FWE-corr^** |
| --- | --- | --- | --- | --- | --- | --- | --- | --- |
| **Main effect nulliparous women and men** | | | | | | | |  |
| 1 | Bilateral lingual gyri  Bilateral cuneus  Bilateral fusiform gyri  Bilateral parahippocampal gyri  Bilateral middle occipital gyri  Bilateral middle temporal gyri  Bilateral inferior occipital gyri  Bilateral posterior cingulate gyrus  Bilateral cerebellum posterior lobe  Bilateral superior occipital gyri  Bilateral precuneus | 18/19/37/17/30/39/36 | 3156 | 30 | -63 | -6 | 121.43 | 0.000 |
| 2 | Right middle frontal gyrus  Right inferior frontal gyrus | 9 | 170 | 39 | 21 | 27 | 36.98 | 0.000 |
| 3 | Left inferior frontal gyrus  Left middle frontal gyrus | 9 | 76 | -39 | 6 | 33 | 31.81 | 0.025 |
| 4 | Bilateral precuneus  Bilateral superior parietal lobule | 7 | 63 | -3 | -66 | 60 | 22.03 | 0.050 |
| 5 | Right cerebellum posterior lobe | - | 66 | 24 | -75 | -24 | 21.67 | 0.043 |
| **Interaction group × condition (happy vs. neutral faces)** | | | | | | |  |  |
| 1 | Left fusiform gyrus  Left parahippocampal gyrus  Left cerebellum posterior lobe | 37/19/36 | 245 | -33 | -30 | -18 | 48.11 | 0.000 |
| 2 | Right parahippocampal gyrus  Right fusiform gyrus  Right cerebellum posterior lobe | 37/19/36 | 216 | 33 | -45 | -9 | 47.35 | 0.000 |
| 3 | Left inferior parietal lobule  Left postcentral gyrus | 40/2 | 107 | -39 | -33 | 42 | 32.76 | 0.006 |
| **Interaction group × condition (sad vs. neutral faces)** | | | | | | | | |
| 1 | Left fusiform gyrus  Left parahippocampal gyrus  Left cerebellum anterior lobe | 37/36 | 199 | -30 | -30 | -21 | 43.93 | 0.000 |
| 2 | Right parahippocampal gyrus  Right fusiform gyrus | 19/37 | 177 | 33 | -45 | -9 | 34.82 | 0.000 |
| 3 | Left postcentral gyrus  Left inferior parietal lobule | 40/2 | 79 | -39 | -30 | 42 | 25.14 | 0.022 |
| **Interaction** **group × condition (happy vs. sad faces)** | | | | | | | | |
|  | None |  |  |  |  |  |  |  |

BA: Brodmann area. The threshold was set at *p* < 0.001 uncorrected at the voxel wise level and *p* < 0.05 with FWE correction at the cluster level.

**Table S2. Functional brain imaging results for the main effects of group and the group by emotional infant faces condition interaction (without grey matter volume as regression).**

| **Clusters** | **Brain regions** | **BA** | **voxels** | **X** | **Y** | **Z** | **F** | ***P*^FWE-corr^** |
| --- | --- | --- | --- | --- | --- | --- | --- | --- |
| **Main effect of group** | | | | | | |  |  |
| 1 | Bilateral lingual gyri  Bilateral cuneus  Bilateral middle occipital gyri  Bilateral fusiform gyri  Bilateral parahippocampal gyri  Bilateral inferior occipital gyri  Bilateral middle temporal gyri  Bilateral cerebellum posterior lobe  Bilateral superior occipital gyri  Bilateral precuneus  Bilateral posterior cingulate gyri  Bilateral superior temporal gyrus | 18/19/17/37/39/20/36/30 | 3476 | 30 | -63 | -6 | 138.25 | 0.001 |
| 2 | Right middle frontal gyrus  Right inferior frontal gyrus | 46/9/6 | 316 | 39 | 21 | 27 | 48.46 | 0.001 |
| 3 | Left inferior parietal lobule  Left precuneus | 7 | 86 | -27 | -51 | 54 | 45.51 | 0.016 |
| 4 | Left inferior frontal gyrus  Left middle frontal gyrus | 9 | 134 | -39 | 6 | 33 | 41.63 | 0.002 |
| 5 | Right brainstem  Right thalamus | - | 149 | -3 | -30 | -3 | 37.80 | 0.001 |
| 6 | Left insula  Left precentral gyrus  Left superior temporal gyrus | 13/22/6 | 118 | -45 | 3 | 0 | 28.30 | 0.004 |
| 7 | Right cingulate gyrus | - | 66 | 18 | -27 | 18 | 26.83 | 0.042 |
| 8 | Right insula  Right precentral gyrus  Right superior temporal gyrus | 13/6 | 77 | 45 | -6 | 12 | 26.47 | 0.024 |
| 9 | Right precuneus  Right inferior parietal lobule  Right superior parietal lobule | 7 | 99 | 27 | -51 | 54 | 22.46 | 0.008 |
| **Interaction group × condition (Happy vs. neutral faces)** | | | | | | |  |  |
| 1 | Left fusiform gyrus  Left parahippocampal gyrus  Left cerebellum posterior lobe | 37/36/19 | 244 | -33 | -30 | -18 | 48.36 | 0.001 |
| 2 | Right parahippocampal gyrus  Right fusiform gyrus | 37/19/36 | 217 | 33 | -45 | -9 | 47.42 | 0.001 |
| 3 | Left inferior parietal lobule  Left postcentral gyrus | 40/2 | 109 | -39 | -33 | 42 | 32.83 | 0.005 |
| **Interaction group × condition (Sad vs. neutral faces)** | | | | | | | | |
| 1 | Left fusiform gyrus  Left parahippocampal gyrus  Left cerebellum anterior lobe | 37/36 | 201 | -30 | -30 | -21 | 44.30 | 0.001 |
| 2 | Right parahippocampal gyrus  Right fusiform gyrus | 19/37 | 174 | 33 | -45 | -9 | 34.96 | 0.001 |
| 3 | Left postcentral gyrus  Left inferior parietal lobule | 40/2 | 80 | -39 | -30 | 42 | 25.28 | 0.002 |
| **Interaction group × condition (Happy vs. sad faces)** | | | | | | | | |
|  | None |  |  |  |  |  |  |  |

BA: Brodmann area. The threshold was set at *p* < 0.001 uncorrected at the voxel wise level and *p* < 0.05 with FWE correction at the cluster level.

[1] YAN C G, WANG X D, ZUO X N, et al. DPABI: Data Processing & Analysis for (Resting-State) Brain Imaging [J]. Neuroinformatics, 2016, 14(3): 339-51.
